# Association of genetic variants with autism spectrum disorder in Japanese children revealed by targeted sequencing

**DOI:** 10.3389/fgene.2024.1352480

**Published:** 2024-08-30

**Authors:** Yuka Shiota, Tomoaki Nishiyama, Shigeru Yokoyama, Yuko Yoshimura, Chiaki Hasegawa, Sanae Tanaka, Sumie Iwasaki, Mitsuru Kikuchi

**Affiliations:** ^1^ Japan Society for the Promotion of Science, Tokyo, Japan; ^2^ Research Center for Child Mental Development, Kanazawa University, Kanazawa, Japan; ^3^ Research Center for Experimental Modeling of Human Disease, Kanazawa University, Kanazawa, Japan; ^4^ United Graduate School of Child Development, Osaka University, Kanazawa University, Hamamatsu University School of Medicine, Chiba University, and University of Fukui, Kanazawa, Japan; ^5^ Institute of Human and Social Sciences, Kanazawa University, Kanazawa, Japan; ^6^ Department of Psychiatry and Neurobiology, Graduate School of Medical Science, Kanazawa University, Kanazawa, Japan

**Keywords:** autism spectrum disorder, genetic architecture, high-functioning autism, next-generation sequencing, single-nucleotide polymorphism, common variant, social responsiveness scale

## Abstract

**Introduction:**

Autism spectrum disorders (ASD) represent a heterogeneous group of neurodevelopmental disorders with strong genetic predispositions. Although an increasing number of genetic variants have been implicated in the pathogenesis of ASD, little is known about the relationship between ASD-associated genetic variants and individual ASD traits. Therefore, we aimed to investigate these relationships.

**Methods:**

Here, we report a case-control association study of 32 Japanese children with ASD (mainly with high-functioning autism [HFA]) and 36 with typical development (TD). We explored previously established ASD-associated genes using a next-generation sequencing panel and determined the association between Social Responsiveness Scale (SRS) T-scores and intelligence quotient (IQ) scores.

**Results:**

In the genotype-phenotype analyses, 40 variants of five genes (*SCN1A*, *SHANK3, DYRK1A, CADPS,* and *SCN2A*) were associated with ASD/TD phenotypes. In particular, 10 *SCN1A* variants passed permutation filtering (false discovery rate <0.05). In the quantitative association analyses, 49 variants of 12 genes (*CHD8, SCN1A, SLC6A1, KMT5B, CNTNAP2, KCNQ3, SCN2A, ARID1B, SHANK3, DYRK1A, FOXP1, and GRIN2B*) and 50 variants of 10 genes (*DYRK1A, SCN2A, SLC6A1, ARID1B, CNTNAP2, SHANK3, FOXP1, PTEN, SCN1A, and CHD8*) were associated with SRS T- and IQ-scores, respectively.

**Conclusion:**

Our data suggest that these identified variants are essential for the genetic architecture of HFA.

## 1 Introduction

Autism spectrum disorder (ASD) is a common neurodevelopmental disorder characterized by impaired social communication and restricted, repetitive behaviors or interests (Masi et al., 2017; Lord et al., 2018; Hirota and King, 2023). In the US, ASD affects approximately 2.3% of children aged 8 years (Hirota and King, 2023). One important aspect of ASD is its heterogeneous clinical features. For example, some individuals exhibit intellectual disability (ID) and limited language ability, while others display motor impairments (Hirota and King, 2023). This heterogeneity is attributed to multiple genetic variations and environmental factors (Baron-Cohen, 2017; Bai et al., 2019).

Genetic variations are associated with varying allelic effect sizes and population frequencies. In addition, genetic variations exist in a continuum, ranging from single nucleotide changes to genomic changes at the chromosomal level. Small-scale variations include single-nucleotide polymorphisms (SNPs) and insertions or deletions (indels) of a short DNA sequence, while typical large-scale variations include copy number variations (CNVs) and chromosomal rearrangement (Sebat et al., 2007). In general, rare variants tend to have a larger effect size than common variants (Grove et al., 2019). The presence of numerous variants with different effect sizes and allele frequencies, along with their interactions with environmental factors, generates a highly complex genetic architecture of ASD (Timpson et al., 2018). Although whole-genome and whole-exome next-generation sequencing (NGS) have been utilized for comprehensive analyses of such polygenic disorders, these approaches are relatively expensive (Mellone et al., 2022). Targeted panel sequencing allows the efficient and accurate detection of variants with high sensitivity (Mellone et al., 2022).

To date, several large cohort studies have identified an increasing number of ASD-associated genetic variants. More than one thousand ASD-associated rare variants have been identified using whole-genome sequencing and transmission and *de novo* association tests (Murtaza et al., 2022). By exome analyses in a Japanese population, Kimura et al. (2022) identified rare synaptic function-related variants. However, these large-scale cohort studies might have included individuals with various degrees of autistic traits and severity of ID. Moreover, relationships between genetic variants and individual ASD traits, such as speech development, social responsiveness, and intelligence remain largely unexplored.

In this study, we performed case-control NGS analyses combined with psychological assessments on Japanese children with ASD, mainly with high-functioning autism (HFA). As HFA is not considered to be a distinct and absolute diagnostic category, we assessed it as a form of ASD, which was highly heterogeneous in phenotype. Children with ASD with an intelligence quotient (IQ) of 70 or above were defined as having HFA (Alvares et al., 2020). Using short-read-based methods, we analyzed SNPs and short indels, regardless of whether they were rare or common in population frequency. We aimed to further our understanding of ASD by clarifying the relationship between ASD-associated variants and individual ASD traits.

## 2 Materials and methods

### 2.1 Participants

Participants, all of whom lived in the Hokuriku District of Japan, were called upon to participate in the Bambi Plan at Kanazawa University and its affiliated hospitals. The participants were children diagnosed with ASD and typically developing (TD) children who did not exhibit apparent developmental delay. Additionally, those with low birth weight were referred to participate in the Bambi Plan by the Department of Pediatrics of Kanazawa University Hospital. The children were 5–8 years old and were able to sit for 1 h, which is a requirement for the IQ assessment as it must be taken seated. Children with low birth weight and who exhibited developmental delay were assessed with the assessments, which were established according to the Diagnostic and Statistical Manual of Mental Disorders (fourth edition) (American Psychiatric Association, 2000) using the Diagnostic Interview for Social and Communication Disorders (Wing et al., 2002), Autism Diagnostic Observation Schedule-Generic (Lord et al., 2000), and Autism Diagnostic Observation Schedule 2 (Lord C et al., 2012), as well as children with ASD. Those who did not fully meet the criteria of these assessments were regarded as individuals with sub-threshold autistic traits (Shiota et al., 2021). In addition, children enrolled in TD were referred to as TD without the formal assessments. IQ was assessed using the mental processing composite scale of the Kaufman Assessment Battery for Children (K-ABC) (Kaufman AS, 1983). Overall, 118 children with ASD (92 males, 26 females), 104 with TD (59 males, 45 females), 3 with sub-threshold autistic traits (3 males), and 10 with low birth weight (3 males, 7 females) were enrolled (Supplementary Figure S1).

### 2.2 Target gene regions for enrichment

Based on the SFARI gene database (https://gene.sfari.org/) and previous reports (Satterstrom et al., 2020; Fóthi et al., 2022; Murtaza et al., 2022; Qiu et al., 2022), 16 highly confident ASD-associated genes, one promoter region, and 20 intergenic regions containing ASD-associated SNPs were selected for the biotinylated oligonucleotide probe design for enrichment (Table 1).

**TABLE 1:** Genes and SNPs targeted in this study.

No.	ASD-associated genes	Chromosome	Description	References
1	*POGZ*	1q21.3	Binds to HP1-α in a competitive manner with PxVxL motif-binding proteins such as TIF1-beta and INCENP. Knockdown in human cell lines caused mitotic defects, with accelerated mitosis, abnormal chromosome segregation, nuclear fragmentation, and disrupted mitotic HP1-α localization and Aurora kinase B activity (Nozawa et al., 2010)	Neale et al., 2012; Iossifov et al., 2012; Iossifov et al., 2014; De Rubeis et al., 2014; Iossifov et al., 2015; White et al., 2016; Stessman et al., 2016; Zhou et al., 2022
2	*SCN1A*	2q24.3	Encodes the large α-subunit of the vertebrate voltage-gated sodium channel essential for the generation and propagation of action potentials (Goldin et al., 1986; Isom, 2002)	Weiss et al., 2003; O'Roak et al., 2011; O'Roak et al., 2012a; O'Roak et al., 2012b; De Rubeis et al., 2014; Yuen et al., 2017; Ouss et al., 2019; Satterstrom et al., 2020
3	*SCN2A*	2q24.3	Encodes the voltage-gated sodium channel Nav1.2, which plays an important role in the initiation and conduction of action potentials. (Wolff et al., 2017)	Weiss et al., 2003; Sanders et al., 2012; Tavassoli et al., 2014; Jiang et al., 2013; De Rubeis et al., 2014; Iossifov et al., 2015; Ben-Shalom et al., 2017; Wolff et al., 2017; Zhou et al., 2022
4	*FOXP1*	3p13	A transcriptional repressor that plays a critical role in monocyte differentiation and macrophage function (Shi et al., 2008)	O'Roak et al., 2011; Meerschaut et al., 2017; Feliciano et al., 2019; Satterstrom et al., 2020; Trelles et al., 2021, Zhou et al., 2022
5	*SLC6A1*	3p25.3	Encodes a γ-aminobutyric acid (GABA) transporter, which removes GABA from the synaptic cleft (Hirunsatit et al., 2009)	Sanders et al., 2012; Carvill et al., 2015; Sanders et al., 2015; Satterstrom et al., 2020; Johannesen et al., 2018; Mermer et al., 2021; Zhou et al., 2022
6	*ARID1B*	6q25.3	A small subset of SWI/SNF complexes, which contain a Swi2/Snf2-related DNA-dependent ATPase and function in the remodeling of chromatin (Hurlstone et al., 2002; Nie et al., 2003)	Nord et al., 2011; Halgren et al., 2012; De Rubeis et al., 2014; Iossifov et al., 2015; Zhou et al., 2022
7	*SYNGAP1*	6p21.32	Encodes a brain-specific synaptic Ras GTPase activating protein that suppresses signaling pathways linked to NMDA receptor-mediated synaptic plasticity and AMPA receptor membrane insertion (Clement et al., 2012; Berryer et al., 2013)	Hamdan et al., 2011; Iossifov et al., 2015; Zhou et al., 2022
8	*CNTNAP2*	7q35-q36.1	Encodes a neuronal transmembrane protein member of the neurexin superfamily involved in neural-glia interactions and clustering of potassium channels in myelinated axons	Arking et al., 2008; Alarcon et al., 2008; Vernes et al., 2008; Li et al., 2010; Whitehouse et al., 2011; Poot, 2014; Shiota et al., 2021; Shiota et al., 2022; Weinschutz et al., 2023
9	*KCNQ3*	8q24.22	A member of the voltage-gated potassium channel family. The KCNQ2 and KCNQ3 channel subunits can coassemble to form a channel with essentially identical biophysical properties and pharmacologic sensitivities to the native M-channel (Wang et al., 1998)	Gilling et al., 2013; Toma et al., 2014; De Rubeis et al., 2014; Satterstrom et al., 2020; Zhou et al., 2022
10	*PTEN*	10q23.31	Encodes a ubiquitously expressed tumor suppressor dual-specificity phosphatase that antagonizes the PI3K signaling pathway through its lipid phosphatase activity and negatively regulates the MAPK pathway through its protein phosphatase activity (Pezzolesi et al., 2007)	Goffin et al., 2001; O'Roak et al., 2012a; O'Roak et al., 2012b; De Rubeis et al., 2014; Frazier et al., 2015; Iossifov et al., 2015; Zhou et al., 2022
11	*SUV420H1* (*also known as KMT5B*)	11q13.2	Functions as histone methyltransferases that specifically trimethylate nucleosomal histone H4 on lysine 20 (K20) (Schotta et al., 2004)	Sanders et al., 2012; Carvill et al., 2015; Sanders et al., 2015; Satterstrom et al., 2020; Johannesen et al., 2018; Mermer et al., 2021; Zhou et al., 2022
12	*GRIN2B*	12p13.1	NMDA receptors are heterotetramers composed of 2 NMDA receptor-1 (NR1 or GRIN1) subunits and 2 NR2 subunits, such as GRIN2B (Matta et al., 2011)	O'Roak et al., 2011; Myers et al., 2011; O'Roak et al., 2012a; De Rubeis et al., 2014; Iossifov et al., 2015; Platzer et al., 2017; Zhou et al., 2022
13	*CHD8*	14q11.2	An ATP-dependent chromatin-remodeling factor that regulates transcription of beta-catenin target genes (Thompson et al., 2008)	O'Roak et al., 2012b; O'Roak et al., 2012a; O'Roak et al., 2012b; Talkowski et al., 2012; Iossifov et al., 2014; De Rubeis et al., 2014; Bernier et al., 2014; Iossifov et al., 2015; Zhou et al., 2022
14	*ADNP*	20q13.13	A homeodomain-containing zinc finger protein with transcription factor activity that is essential for brain formation (Gozes, 2007; Mandel and Gozes, 2007)	O'Roak et al., 2012a; De Rubeis et al., 2014; Helsmoortel et al., 2014; Iossifov et al., 2015; Zhou et al., 2022
15	*DYRK1A*	21q22.13	Encodes a member of the dual-specificity tyrosine phosphorylation-regulated kinase (DYRK) family and participates in various cellular processes. It is a highly conserved gene located in the so-called Down Syndrome critical region (DSCR), a part of chromosome 21 that is responsible for the majority of phenotypic features in Down syndrome (van Bon et al., 2011)	O'Roak et al., 2012b; De Rubeis et al., 2014; Iossifov et al., 2015; van Bon et al., 2016; Evers et al., 2017; Zhou et al., 2022
16	*SHANK3*	22q13.33	Encodes a scaffolding protein that is enriched in postsynaptic densities of excitatory synapses (Yi et al., 2016)	Durand et al., 2007; Gauthier et al., 2010; Leblond et al., 2014; Iossifov et al., 2015; Zhou et al., 2022
**No.**	**Promoter region**	**Chromosome**	**Mapped Gene**	**References**
1	HTTLPR	17q11.2	SLC6A4/5HTT	Devlin et al., 2005; Brune et al., 2006; Velasquez et al., 2017; Wang et al., 2019
**No.**	**SNPs in intergenic regions**	**Location**	**Gene**	**References**
1	rs1620977	1:72263459	*NEGR1*	Grove et al. (2019)
2	rs34213746 (rs201910565)	1:96096255	*RNU1-130P, LINC02790*	Grove et al. (2019)
3	rs1452075	3:62495388	*CADPS*	Grove et al. (2019)
4	rs16854048	4:42121711	*BEND4*	Grove et al. (2019)
5	rs325506	5:104676602	*NIHCOLE, RNU6-334P*	Grove et al., 2019; Baranova et al., 2022
6	rs2388334	6:98143746	*EIF4EBP2P3, MIR2113*	Grove et al. (2019)
7	rs111931861	7:105103772	*KMT2E*	Grove et al. (2019)
8	rs7794745	7:146792514	*CNTNAP2*	Vardarajan et al., 2013; Warrier et al., 2015; Zhang et al., 2019
9	rs10099100	8:10719265	*RP1L1, SOX7*	Grove et al., 2019; Wu et al., 2020
10	rs11787216	8:142615222	*MIR1302-7, C8orf90*	Grove et al. (2019)
11	rs2094530	13:50990321	*GUCY1B2*	Gialluisi et al. (2021)
12	rs10149470	14:103551616	*RNU7-160P, BAG5*	Grove et al. (2019)
13	rs113877277ß	17:38498858	*ARHGAP23*	Price et al. (2020)
14	rs6035856	20:2207896	*LOC388780*	Gialluisi et al. (2021)
15	rs6035857	20:2207898	*LOC388780*	Gialluisi et al. (2021)
16	rs6047381	20:2204711	*LOC388780*	Gialluisi et al. (2021)
17	rs6137325	20:2207297	*LOC388780*	Gialluisi et al. (2021)
18	rs6137326	20:2207298	*LOC388780*	Gialluisi et al. (2021)
19	rs71190156	20:14855610	*MACROD2*	Grove et al., 2019; Wu et al., 2020
20	rs910805	20:21267478	*XRN2, ZNF877P*	Grove et al., 2019; Wu et al., 2020; Peyrot and Price, 2021

### 2.3 Sequencing and data processing

Buccal mucosa was collected using a swab. Genomic DNA was extracted from the buccal mucosa using a Maxwell RSC Instrument (Promega, Madison, WI, United States) with the Maxwell RSC Blood DNA Kit (Promega), according to the manufacturer’s instructions. Subsequently, whole-genome amplification was performed on 147 genomic DNA samples (88 were excluded) using a PicoPLEX WGA kit (TaKaRa Bio, Mountain View, CA, United States), according to the manufacturer’s instructions. An Illumina paired-end sequencing library was constructed using the KAPA HyperPlus Kit (Kapa Biosystems, Wilmington, MA, United States), followed by enrichment of target sites with xGen Custom Hyb Panel (Integrated DNA Technologies) and sequencing using an Illumina iSeq 100 System. Adapter sequences were trimmed from the reads using Trimmomatic v0.39 and mapped to the human reference genome (hg38) using Burrows–Wheeler Aligner v0.7.17 (https://github.com/lh3/bwa). Libraries from the amplified DNA resulted in insufficient sequencing and mapping rates; thus, the data were not used for further analysis. Libraries constructed directly from the unamplified DNA yielded sufficient read-count mapping rates. Variant calls were carried out using HaplotypeCaller in GATK version 4.2.6.1 (McKenna et al., 2010) to obtain the gVCF file for each individual. Further, individual gVCF files were combined with CombineGVCFs (GATK version 4.4.0.0), and final genotype sets were called from the whole cohort of preselected target regions (Supplementary Table S1) using GenotypeGVCFs with 3-bp expansion (command: ‘--interval-padding’) to obtain the cohort genotype VCF file. Cohort genotype VCF files were read with PLINK ver. 1.9 (Purcell et al., 2007) to generate data files named plink.{bed,fam,bim}. Sex data were manually recorded on the. fam file. The phenotype data were encoded as 01 (TD), 02 (ASD), G1 (sub-threshold autistic traits), and d1 (low birth weight) (Supplementary Table S2). Using these files, case-control association tests were performed with PLINK. Genotype associations with IQ scores and Social Responsiveness Scale (SRS) (see Supplementary Material for details) scores, as quantitative phenotypes, were analyzed with PLINK. Statistically significant associations at the variant sites were further investigated using the Integrative Genomics Viewer (Robinson et al., 2011), and the validity was confirmed.

### 2.4 Statistical analyses

Statistical analyses were performed using R version 4.3.0 (R Core Team, 2021). SRS and IQ were treated as continuous variables for the quantitative association analyses. False discovery rates (FDRs) were calculated with the *p. adjust* function in R with the “BH” method, as previously described (Benjamini and Hochberg, 1995).

## 3 Results

### 3.1 Genotyping

Target-enrichment sequencing following whole-genome amplification resulted in low-quality reads. Subsequent direct library construction and enrichment yielded high-quality reads in 79 of the 88 samples. Consequently, genomic DNA from 79 children (32 with ASD, 36 with TD, 3 with sub-threshold autistic traits, and eight children with low birth weight; 53 males and 26 females aged 5–8 years; Supplementary Table S2) were processed for further analyses (Supplementary Figure S1).

### 3.2 Participants’ characteristics

The mean age of the children was 5.6 and 5.4 years for ASD and TD, respectively (Table 2). The sex ratio was biased toward males in participants with ASD (27:5) but comparable in those with TD (20:16). The IQ scores of three children were not obtained. SRS T-scores and IQ scores of these 79 children are plotted in Supplementary Figure S2 with ASD phenotype diagnosis (Supplementary Table S3), showing positive and negative correlation of SRS T-scores and IQ scores with ASD diagnosis, as demonstrated through Welch’s two-sample *t*-test (Table 2).

**TABLE 2:** Participants’ characteristics.

	ASD *N* = 32	TD *N* = 36	*χ* ^*2*^ or *t*	*p*
Age	5.63	5.36	−1.52	0.13
Sex (% Male)	84.3%	55.6%	6.59	0.01
SRS total score	71.76 (14.99)	46.17 (6.36)	−9.29	8.2 × 10^−12^
K-ABC Mental Processing scale	^a^89.65 (17.39)	105.54 (13.86)	4.18	1.0 × 10^−4^

^a^ *N* = 29.

Numbers are mean (standard deviation).

ASD, autism spectrum disorder; K-ABC, kaufman assessment battery for children; SRS, social responsiveness scale; TD, typical development.

### 3.3 Genotype–phenotype association analyses

**FIGURE 1:**
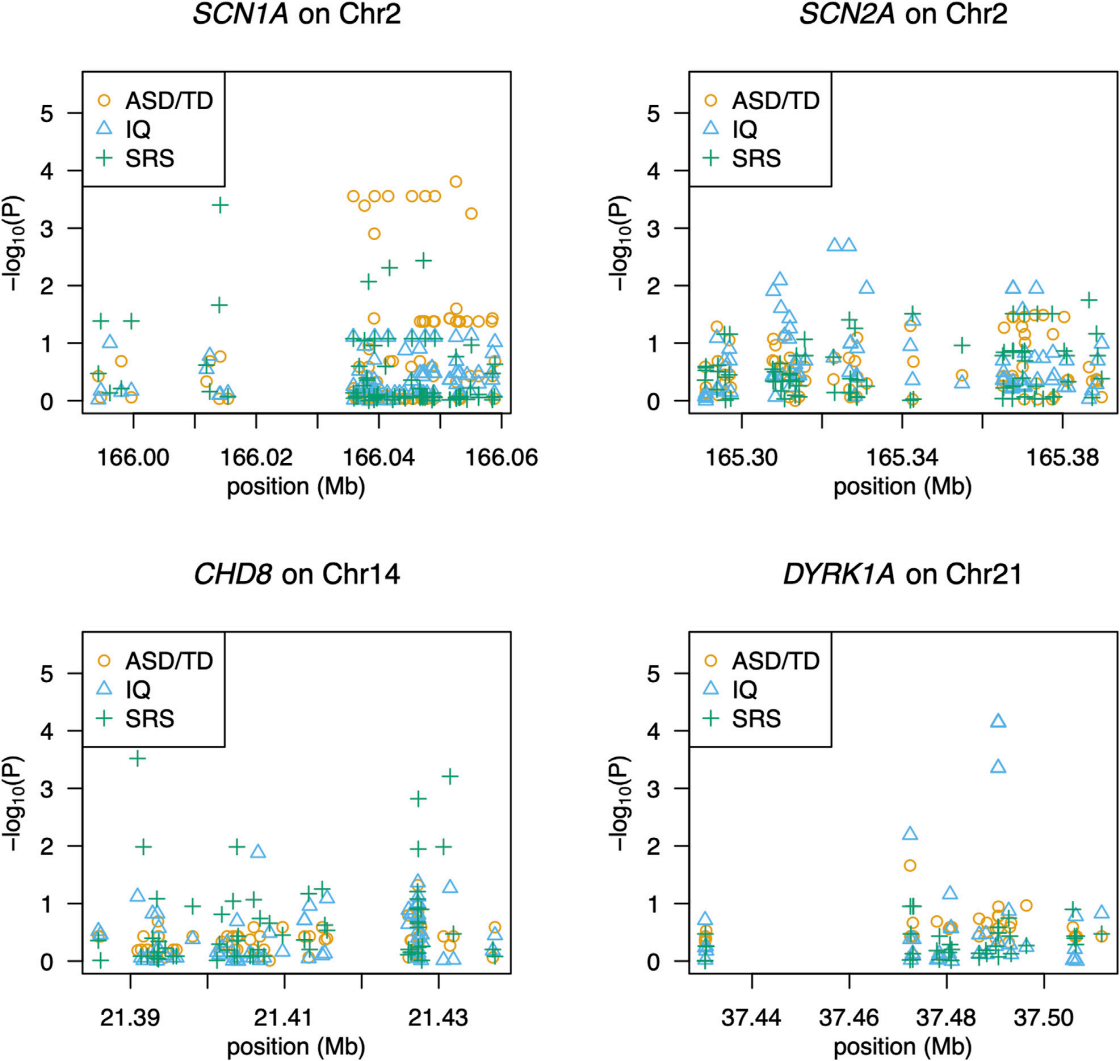
Association of SNPs with ASD, social responsiveness, or intelligence. The gene name and its chromosomal location are given above each panel. The *x*-axis shows the locations of SNPs, and the *y*-axis shows significance as -log_10_(P). Each symbol represents an individual. Circles (in orange), triangles (in blue), and pluses (in green) indicate association with ASD, IQ scores, and SRS-T scores, respectively.

We detected 1,418 variant sites with an average call rate of 70% (Supplementary Data). In total, 748 variants were called at a genotype call rate of 95% (number of individuals = 79). Forty variants were associated with ASD/TD (*p* < 0.05, chi-square tests); low birth weight and sub-threshold autistic traits were not included in the analyses because the autistic phenotypes were not strictly defined. Thus, 68 children were included in the final analyses (Supplementary Figure S1). Among these, variants within the *SCN1A* gene, which encodes the pore-forming α-subunit of the Nav 1.1 voltage-gated sodium channel, exhibited the strongest association with ASD/TD (χ^2^ = 18.8–5.80, *p =*1.45 × 10^-5^-0.016) (Figure 1). In addition, ASD/TD was correlated with variants in *SHANK3* (χ^2^ = 5.84–5.04, *p =* 0.016–0.025), *DYRK1A* (χ^2^ = 4.64, *p =* 0.03)*, CADPS* (χ^2^ = 4.52, *p =* 0.03)*,* and *SCN2A* (χ^2^ = 4.43–3.93, *p =* 0.04–0.05) genes. Compared with *SCN2A* (encodes the pore-forming α-subunit of the Nav 1.2 voltage-gated sodium channel) variants rs2304010, rs10930160, rs2304012, rs767942624, rs1252589686, and rs59934051 (Figure 1), the *SCN1A* variant rs79990586 exhibited lower *p*-values under dominant model (*p =* 0.01–0.03). Although 37.4 sites were expected to result in *p* < 0.05, if 748 sites are independent of ASD, then the associations of 10 variants (dbSNP: Chromosome: BP; rs28663047: 2: 166052559, rs11691603: 2: 166035836, rs10168027: 2: 166039309, rs10198801: 2: 166041507, rs67636132: 2: 166045389, rs11690962: 2: 166047552, rs1021999648: 2: 166049124, rs1187504368: 2: 166037655, rs1541783: 2: 166055076, and rs1019723: 2: 166039251) of *SCN1A* are calculated to have FDRs <0.05 (*q =* 2.8 × 10^-3^-0.02) and permutation test (*p* = 2.1 × 10^-3^-0.04), as summarized in Table 3 (also see Supplementary Table S4 for details). We found that 42 variants were associated with HFA (29 children with HFA) excluding individuals with ASD with IQ < 70/TD (*p* < 0.05, chi-square test), as summarized in Supplementary Table S5.

**TABLE 3:** Association analysis of genotype and ASD.

CHR	Gene	SNP	BP	A1	A2	P	FDR_P	FAM_perm_P	Correl Coeff	SRS_P	IQ_P
2	*SCN1A*	rs28663047	166052559	C	T	1E-05	0.003	0.002	−0.499	0.095	0.035
2	*SCN1A*	rs11691603	166035836	G	A	3E-05	0.003	0.004	−0.539	0.042	0.034
2	*SCN1A*	rs10168027	166039309	G	A	3E-05	0.003	0.004	−0.539	0.042	0.034
2	*SCN1A*	rs10198801	166041507	T	G	3E-05	0.003	0.004	−0.539	0.042	0.034
2	*SCN1A*	rs67636132	166045387	A	AG	3E-05	0.003	0.004	−0.539	0.042	0.034
2	*SCN1A*	rs11690962	166047552	T	G	3E-05	0.003	0.004	−0.539	0.042	0.034
2	*SCN1A*	rs1021999648	166049124	T	TAC​TTT​ACA​GTG​CAA​AGT​ATT​TCT​TCA​TTA​TGA​AGA​AAT​GAC​ATT​AGA​TTA​GAT​ACA​GTG​CAA​AGA​TAC​TTT​AGA​C^a^	3E-05	0.003	0.004	−0.497	0.042	0.034
2	*SCN1A*	rs1187504368	166037655	CTT	CT	4E-05	0.004	0.005	−0.436	0.045	0.048
2	*SCN1A*	rs1541783	166055076	C	T	1E-04	0.008	0.016	−0.486	0.082	0.041
2	*SCN1A*	rs1019723	166039251	A	G	3E-04	0.020	0.039	0.448	0.105	0.171
2	*SCN1A*	rs367905968	166052619	A	AT	1E-03	0.085	0.178	−0.347	0.627	0.088
2	*SCN1A*	rs6432861	166046718	C	T	4E-03	0.119	0.467	−0.317	0.640	0.186
2	*SCN1A*	rs13383628	166047150	T	C	4E-03	0.119	0.467	−0.317	0.640	0.186
2	*SCN1A*	rs11690959	166047515	G	A	4E-03	0.119	0.467	−0.317	0.640	0.186
2	*SCN1A*	rs1542484	166048865	A	G	4E-03	0.119	0.467	−0.317	0.640	0.186
2	*SCN1A*	rs1542483	166049062	T	A	4E-03	0.119	0.467	−0.317	0.640	0.186
2	*SCN1A*	rs1841546	166052594	C	T	4E-03	0.119	0.467	−0.317	0.640	0.186
2	*SCN1A*	rs3812718	166053034	C	T	4E-03	0.119	0.467	−0.317	0.640	0.186
2	*SCN1A*	rs2217199	166053185	A	G	4E-03	0.119	0.467	−0.317	0.640	0.186
2	*SCN1A*	rs66512822	166054359	ATGTG	A	4E-03	0.119	0.467	−0.317	0.578	0.200
2	*SCN1A*	rs1461203	166056247	C	T	4E-03	0.119	0.467	−0.317	0.640	0.186
2	*SCN1A*	rs7564306	166058385	A	G	4E-03	0.119	0.467	−0.317	0.640	0.186
2	*SCN1A*	rs6147014	166039198	C	CTGAG	4E-03	0.119	0.483	0.301	0.708	0.336
2	*SCN1A*	rs6706163	166051520	C	A	4E-03	0.119	0.483	0.301	0.708	0.336
2	*SCN1A*	rs6750294	166051603	A	T	4E-03	0.119	0.483	0.301	0.708	0.336
2	*SCN1A*	rs8191987	166058504	G	A	4E-03	0.119	0.483	0.301	0.708	0.336
22	*SHANK3*	rs148502223	50676362	T	C	2E-02	0.403	0.930	0.299	0.015	0.642
2	*SCN1A*	rs79990586	166038417	C	T	2E-02	0.403	0.940	0.168	0.525	0.783
22	*SHANK3*	rs12483981	50703953	A	G	2E-02	0.599	0.987	0.243	0.175	0.881
21	*DYRK1A*	rs17229402	37472458	A	T	3E-02	0.610	0.998	0.265	1.000	0.006
3	*CADPS*	rs1452075	62495388	C	T	3E-02	0.610	0.999	−0.253	0.422	0.461
2	*SCN2A*	rs2304010	165367537	A	G	4E-02	0.610	0.999	−0.249	0.191	0.986
2	*SCN2A*	rs10930160	165367696	A	G	4E-02	0.610	0.999	−0.249	0.191	0.986
2	*SCN2A*	rs2304012	165370468	G	A	4E-02	0.610	0.999	−0.249	0.191	0.986
2	*SCN2A*	rs767942624	165380360	T	TG	4E-02	0.610	0.999	−0.249	0.191	0.986
2	*SCN2A*	rs12614399	165293790	C	G	4E-02	0.610	0.999	−0.229	0.900	0.045
2	*SCN2A*	rs12692768	165372999	T	C	4E-02	0.610	1.000	0.224	0.883	0.113
2	*SCN2A*	rs1368238	165375124	G	A	4E-02	0.610	1.000	0.224	0.883	0.113
2	*SCN2A*	rs2304015	165307838	A	G	4E-02	0.610	1.000	0.259	0.122	0.009
2	*SCN2A*	rs62174667	165342016	G	A	5E-02	0.610	1.000	−0.226	0.769	0.069

CHR, chromosome; SNP, SNP ID; BP, Physical position (base-pair); A1, Minor allele name (based on whole sample); A2, major allele name.

P, Asymptotic *p*-value for this test; P_dom, under dominant model; FDR_P, FDR *p*-value; FAM_perm_P, Family-wise permutation test *p*-value.

Correl Coeff, correlation coefficient; SRS_P, association with SRS T-scores *p*-value; IQ_P, association with IQ, scores *p*-value; NA, not applicable.

^a^ Common alleles are T>C.

### 3.4 Association with SRS T-scores

Quantitative association analyses revealed 49 associated variants (*p =* 9.7 × 10^-4^-0.05; Supplementary Table S6). The strongest association (*p =* 9.7 × 10^-4^-0.02) was found in variants of the *CHD8* gene, which encodes chromodomain helicase DNA-binding protein 8 (Figure 1). Additionally, rs148502223 (*SHANK3*; SH3 and multiple ankyrin repeat domains 3) was more closely associated with ASD than with TD (χ^2^ = 5.84, *p =* 0.02). Welch’s two-sample *t*-test revealed that individuals with the C/T genotype at rs148502223 had higher mean SRS T-scores than those with the C/C genotype (*p =* 0.01328). However, this association with rs148502223 and all detected variants was not significant after multiple-testing correction (FDRs >0.05).

### 3.5 Association with IQ scores

Using quantitative association analyses, we identified 50 variants (*p =* 3.0 × 10^-5^-0.05; Supplementary Table S7). The variants with the strongest association were *DYRK1A* (*p =* 3.0 × 10^-5^-6.3 × 10^−3^)*,* which encodes dual-specificity tyrosine-(Y)-phosphorylation regulated kinase 1A (Figure 1). rs17229402 of *DYRK1A* was associated with IQ scores (ASD vs. TD, *p =* 0.0313). Although the association of seven *DYRK1A* variants with IQ scores had FDR <0.05, (*q =* 4.4 × 10^-3^-0.02) (Supplementary Table S6), we did not detect reads through BAM file inspection, to support the presence of the variants.

### 3.6 Variants showing association with multiple phenotypes

Associations with both ASD/TD and SRS T-scores were observed in seven *SCN1A* variants (SNPs: rs11691603, rs10168027, rs10198801, rs11690962, and rs1187504368; indel: rs67636132 and rs1021999648) and one *SHANK3* SNP (rs148502223) (Table 3). Nine *SCN1A* variants (SNPs: rs28663047, rs11691603, rs10168027, rs10198801, rs67636132, rs11690962, rs1187504368, and rs1541783; indel: rs1021999648), one *DYRK1A* SNP (rs17229402), and two *SCN2A* SNPs (rs12614399 and rs2304015) showed association with both ASD/TD and IQ scores (Table 3). One *CHD8* SNP (rs10467770), six *SCN1A* SNPs (rs11691603, rs10168027, rs10198801, rs11690962, and rs1187504368; indel: rs1021999648) and one unconfirmed *SCN2A* (BP: 166045387 on Chromosome 2) showed association with both SRS T-scores and IQ scores (Table 3).

## 4 Discussion

In our cohort, mainly containing children with HFA, we identified 40 variants within five genes that are associated with ASD. In addition, we observed an association of 49 and 50 variants including *CHD8* and *DYRK1A* with SRS T-scores and IQ scores, respectively. With the exception of seven unregistered *DYRK1A* variants and one unregistered *SCN2A* variant, all other identified variants were reported in dbSNP (Table 3; Supplementary Tables S5, S6). In particular, 10 variants of *SCN1A* passed filtering of the permutation test and the FDR was below 0.05, as determined through BH correction. These data provide some insights into the genetic architecture of HFA.

Of the variants found to be associated with the phenotypes, none were present in exons; all were found in introns and predicted to be benign variations that are not expected to cause medically important phenotypes. Given the nature of association studies, variants may constitute a linked haplotype group that results in improper expression levels in specific cell types. Confirmation of the expression levels is not possible in humans and is beyond the scope of the current report. Notably, HFA may be caused by a small effect size rather than a large effect size of the major ASD genes; that is, the genes are highly functional because gene function is only subtly impaired. The cumulative impact of hundreds of risk alleles from common variations (both SNPs and CNVs) has been observed to lower penetrance effects in HFA (Toma, 2020). A large genome-wide association study reported that the polygenic contribution of common SNPs may be more prominent in patients with HFA than in patients with both ASD and ID (Grove et al., 2019). Thus, the accumulation of genetic SNPs with small effect sizes may contribute to HFA. All ASD-related variations were already in dbVAR and are known polymorphisms with little impact on fitness. Given that the subtle effects of intronic variation may be linked to the phenotype, it may be worth designing probes on introns and flanking regions in addition to those in the exons of the candidate target genes.

However, in our cohort, variants in *SCN2A* were associated with ASD, and those on *SCN1A* were most strongly associated with the ASD phenotype. Among them, SNP rs3812718 is a known risk factor for epilepsy (Tang et al., 2014; Wang et al., 2018; Zhi et al., 2018). *SCN1A* and *SCN2A*, both of which are located on chromosome 2, encode voltage-gated sodium channel α-subunits Nav1.1 and Nav1.2, respectively. Both proteins play important roles in the initiation and propagation of action potentials, thereby modulating the neural network activity (Barbieri et al., 2023). Missense mutations in these genes have been identified in inherited seizure disorders, including generalized epilepsy with febrile seizures plus (GEFS+) (Meisler et al., 2001), as well as in ASD (O'Roak et al., 2011; Reynolds et al., 2020). Loss-of-function mutations in *SCN1A* have been observed in most cases of Dravet syndrome (DS), manifesting as epileptic seizures, hyperactivity, autistic traits, and cognitive decline. Loss of function of the Nav1.1 channel from familial febrile seizures to GEFS+ and finally DS results in a progressive and severe DS phenotype (Catterall, 2014; Ishii et al., 2017). In our study, no individuals had a clinical history of epilepsy (unpublished data), and all variants were detected in introns. A possible explanation is that these intronic variants, which do not destroy the channel structure, may contribute only to autistic traits in HFA but not to ASD with epileptic seizures.

One key aspect of the heterogeneity of ASD may result from diverse IQ (Wolff et al., 2022). In clinical reports of ASD cases, more individuals with ASD had above-average IQ and fewer had below-average IQ, with the latter including -ID (Mayes and Calhoun, 2003; Rommelse et al., 2015). Autistic traits can be found and examined in any type of psychiatric or neurodevelopmental disorder, as well as in individuals without a disorder (Mottron and Bzdok, 2020). This could account for the increase in reports of individuals with ASD with an average or above-average IQ (Wolff et al., 2022).

The cumulative effect of diverse common risk alleles from common variants is thought to cause HFA (Toma, 2020). For example, 22 rare SNPs were documented in delayed speech development in Spanish children with HFA (Alvarez-Mora et al., 2016). Nakata et al. (2019) found that the downregulation of miR-6126 in HFA was correlated with the severity of social deficits. More recently, Wang et al. (2023) noted that ASD-associated genes were responsible for impairments in social communication but not in cognitive functions. Individuals with HFA carried fewer disruptive *de novo* variants than those with both ASD and ID (Wang et al., 2023). These observations imply that HFA-related genes are distinct from low-functioning autism-related genes. In addition, nine new ASD-associated genes were revealed, including *SLC35G1*, in which they identified recurrent mutations in HFA probands (Wang et al., 2023). In the future, an HFA group should be compared carefully with an ASD + ID group to identify HFA-specific factors.

We also found that *CHD8* variants were strongly associated with SRS T-scores. The protein product of *CHD8*, one of the major ASD-associated genes, functions as a chromatin-remodeling factor that regulates the expression of many genes including those for β-catenin and several components of the p53 pathway (Krumm et al., 2014; Katayama et al., 2016). A large cohort of a two-stage analysis of rare *de novo* and inherited coding SNPs identified *CHD8* as a gene with exome-wide significance (*p* < 2.5 × 10^−6^) (Zhou et al., 2022). Furthermore, *CHD8* regulates the expression of ASD-associated genes related to synaptic function and neurodevelopment during neurogenesis (Sugathan et al., 2014; Cotney et al., 2015; Paulsen et al., 2022). *DYRK1A* variants were strongly associated with IQ scores. *DYRK1A* encodes a dual-specificity tyrosine phosphorylation-regulated kinase 1A that contributes to neural development by phosphorylating various substrates including transcription, splicing, and synaptic proteins (Park et al., 2009). *DYRK1A* has also been implicated in the development of intelligence (Earl et al., 2017; Neumann et al., 2018; Trost et al., 2022); however, the site variations discovered in the present analysis have not been confirmed through alignment.

Both SRS T-score and IQ are important criteria for the classification of ASD. However, it remains unclear whether the same genes are associated with both of them. Previous studies have reported a relationship between IQ and autistic traits. Bölte et al. (2008) reported a low correlation between SRS and IQ. In contrast, Marinopoulou et al. (2023) demonstrated that intellectual functioning was negatively associated with SRS in children with ASD. Presumably, these SNPs detected in this study may independently affect both social responsiveness and intelligence through complex interactions.

This study has a few limitations. First, the sample size was small. To detect the small-effect size genes by high-multiplicity comparison, a sample size of a few thousand would be necessary. Second, variants were determined as substitutions or small indels, and such SNPs were not phased; thus, the haplotype of the entire gene structure was not determined. Haplotypes can be reconstructed using long-read sequencing or family genetic analysis. Finally, a targeted panel was constructed by the combination of highly-confident ASD-associated genes known at the beginning of this study. Optimizing the NGS panel by incorporating new variants and/or by removing inappropriate ones would enable the precise elucidation of genetic architectures of different subtypes of ASD.

In summary, our results indicate the presence of core HFA-associated genes in the Japanese population. Future studies, combined with a trio analysis of children and their parents at a larger scale, would define in detail the genetic architecture of HFA.

## Data Availability

The data presented in the study are deposited in the NBDC repository, accession number JGAS000731.
