# Supplementary material for: Association of genetic variants with autism spectrum disorder in Japanese children revealed by targeted sequencing
Source: Front Genet. 2024 Aug 30;15:1352480. doi: 10.3389/fgene.2024.1352480 (PMC11395840; doi:10.3389/fgene.2024.1352480)
Supplement: Supplementary file 1 [file DataSheet2.pdf]

Supplementary Materials

1 Supplementary Data

Data processing

Case-control association tests were performed using PLINK (Purcell et al., 2007) with *plink --pheno asdphenotype2.txt --bfile plink --assoc --geno 0.05 --out cohort79_withsex* with or without dominance specification. The text file *asdphenotype2.txt* records the phenotypes of all genotyped participants (Supplementary Table 2). Additionally, case-control full model association tests were performed using *plink --pheno asdphenotype2.txt --bfile plink --model --geno 0.05 --out cohort79_withsex.* Genotype associations with IQ and SRS (see Assessment of social reciprocity for details) scores were analyzed using *plink --pheno iq.txt --bfile cohort79 --assoc --geno 0.05 --out cohort79_withsex_iq* and *plink --pheno srsscore2.tsv --bfile cohort79 --assoc --geno 0.05 --out cohort79_withsex_srs,* respectively. Family-wise *p*-values were obtained using PLINK permutation procedures with *plink --pheno asdphenotype2.txt --bfile cohort79 --assoc mperm=1e8 --out asd79_mperm --geno 0.05*.

Assessment of social reciprocity

The SRS (Constantino and Gruber, 2005) scores were used to assess social reciprocity. This is a validated quantitative measure for characterizing traits and symptoms of ASD (Constantino et al., 2003). It measures the sub-scales of social awareness, social cognition, social communication, social motivation, and autistic mannerisms to generate a single measure. We used gender-normed social responsiveness T-scores for each sub-scale (Hus et al., 2013). Higher scores indicate greater difficulties in social reciprocity.

2 Supplementary Figure and Table

2.1 Supplementary Figure

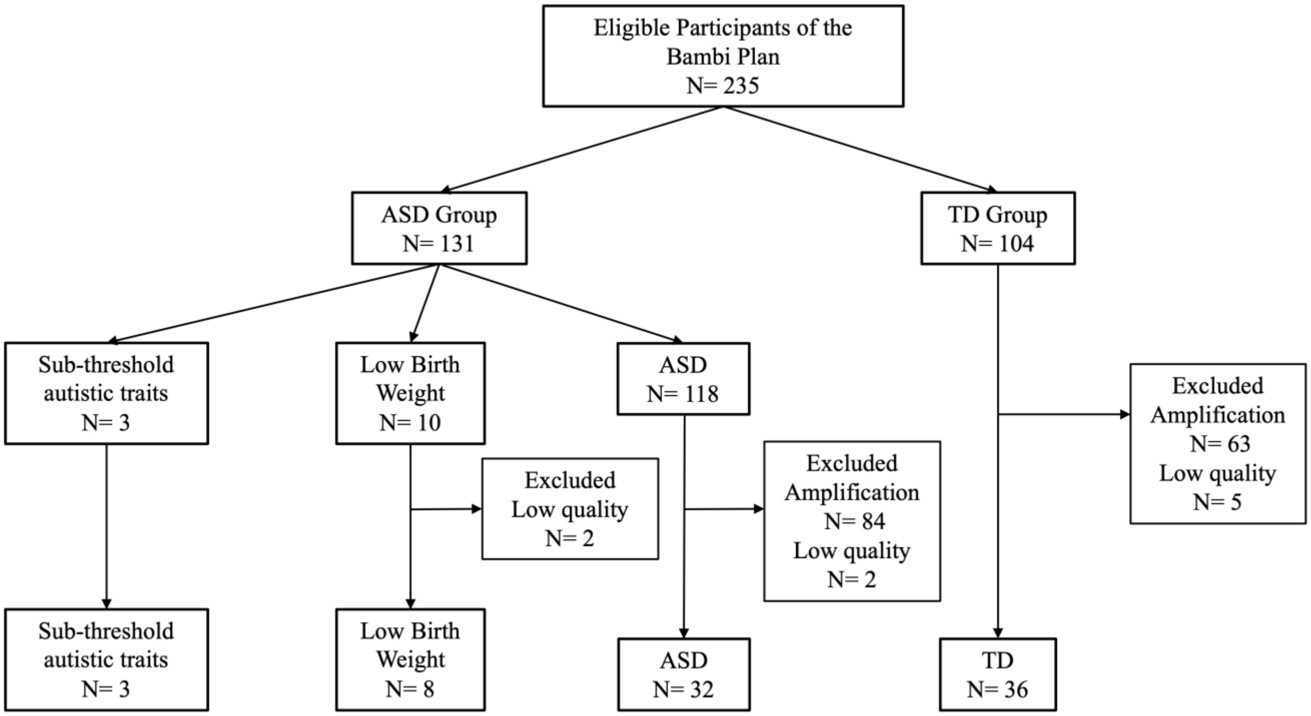

Supplementary Figure 1. Flow diagram of participants

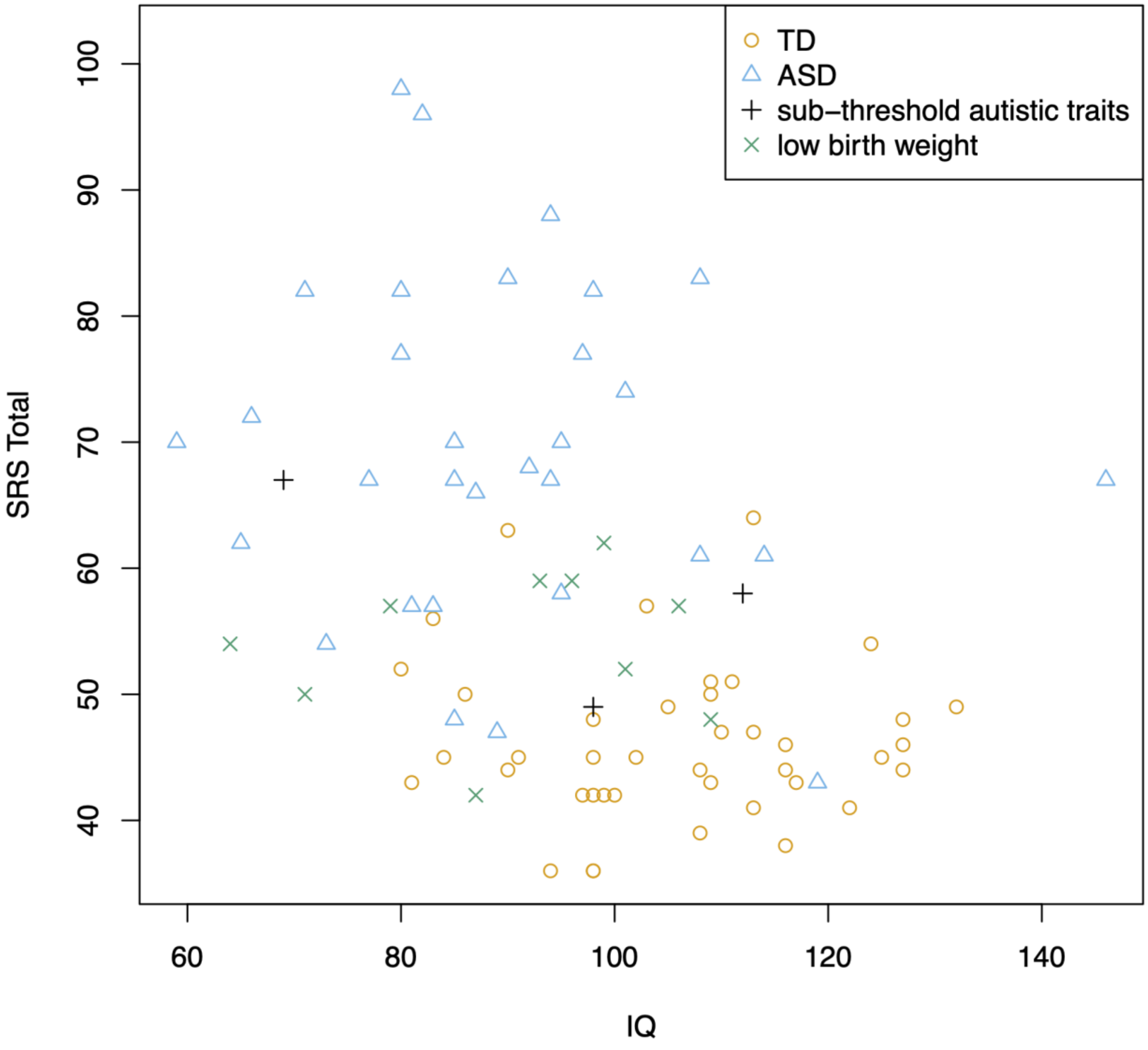

Supplementary Figure 2. SRS T-score and IQ of ASD/TD phenotype. The scatter plot shows the distribution of the SRS-Total and IQ scores within the study cohort. The orange circles represent children with TD. The blue triangles represent children with ASD. The black cross represents children in the grey zone. The green diagonal crosses represent children with a low birth weight.

2.2 Supplementary Table

Supplementary Table S1. Preselected target regions

Chromosome	Start[a]	End[b]	Gene or SNP
1	151403183	151442204	*POGZ*
2	165991245	166073621	*SCN1A*
2	165239615	165389824	*SCN2A*
3	70959247	71198381	*FOXP1*
3	11017212	11036966	*SLC6A1*
6	156777981	157207891	*ARID1B*
6	33420356	33451906	*SYNGAP1*
7	146116877	148415616	*CNTNAP2*
8	132129262	132480532	*KCNQ3*
10	87864469	87965472	*PTEN*
11	68157688	68190076	*SUV420H1*
12	13562783	13866226	*GRIN2B*
14	21385613	21437298	*CHD8*
20	50890908	50904001	*ADNP*
21	37420375	37512531	*DYRK1A*
22	50674642	50731215	*SHANK3*
1	72263458	72263459	rs1620977
1	96096249	96096250	rs34213746

3	62495387	62495388	rs1452075
4	42121710	42121711	rs16854048
5	104676601	104676602	rs325506
6	98143745	98143746	rs2388334
7	105103771	105103772	rs111931861
7	146792513	146792514	rs7794745
8	10719264	10719265	rs10099100
8	141605121	141605122	rs11787216
13	50990320	50990321	rs2094530
14	103551615	103551616	rs10149470
17	38498857	38498858	rs113877277
20	2207895	2207896	rs6035856
20	2207897	2207898	rs6035857
20	2204710	2204711	rs6047381
20	2207296	2207297	rs6137325
20	2207297	2207298	rs6137326
20	14855597	14855598	rs71190156
20	21267477	21267478	rs910805
17	30236955	30237505	HTTLPR

a. Start position (left end) of the region on the hg38 reference genome. The count starts from 0 according to the BED format. b. End position (right end) of the region. The length of the region is (End - Start).

Supplementary Table S2. Phenotype encoding for association analysis

Phenotype	Number of cases enrolled	Number of cases in the final analysis	code
TD	104	36	01
ASD	118	32	02
Sub-threshold autistic traits	3	3	G1
Low birth weight	10	8	d1

Supplementary Table S3. Sub-scores of ADOS-G or ADOS-2 in children with ASD

	Module 1<br>ASD N = 5	Module 2<br>ASD N = 9	Module 3<br>ASD N = 1
**ADOS-G sub-scale**			
Communication	5.20 (0.75)	4.56 (1.77)	4.00 (0)
Interaction	9.80 (2.64)	8.11 (1.37)	9.00 (0)
Total (Communication + Interaction)	15.00 (2.90)	12.67 (2.98)	13.00 (0)
Imagination / Creativity	3.40 (1.20)	1.00 (0.67)	0.00 (0)
Stereotypic Behavior / Restricted Interest	2.60 (1.20)	1.56 (0.68)	1.00 (0)
	Module 1<br>ASD N = 1	Module 2<br>ASD N = 15	Module 3<br>ASD N = 1
**ADOS-2 sub-scale**			
Communication	2.00 (0)	1.54 (0.81)	2.00 (0)
Interaction	15.80 (0)	7.20 (2.04)	5.00 (0)
Total (Communication + Interaction)	17.00 (0)	8.73 (2.64)	7.00 (0)
Repetitive and Restricted Behavior	7.00 (0)	2.20 (1.72)	2.00 (0)

Numbers are mean (standard deviation) or counts. ADOS-G, Autism Diagnostic Observation Schedule-Generic; AOS-2, Autism Diagnostic Observation Schedule 2; ASD, autism spectrum disorder.

Supplementary Table S4. Association analysis of genotype and ASD

CHR	Gene	SNP	BP	A1	F_A	F_U	A2	P	P_dom	FDR_P	FAM_perm_P
2	*SCN1A*	rs28663047	166052559	C	0.063	0.375	T	1.5E-05	NA	0.003	0.002
2	*SCN1A*	rs11691603	166035836	G	0.063	0.361	A	2.8E-05	NA	0.003	0.004
2	*SCN1A*	rs10168027	166039309	G	0.063	0.361	A	2.8E-05	NA	0.003	0.004
2	*SCN1A*	rs10198801	166041507	T	0.063	0.361	G	2.8E-05	NA	0.003	0.004
2	*SCN1A*	rs67636132	166045387	A	0.063	0.361	AG	2.8E-05	NA	0.003	0.004
2	*SCN1A*	rs11690962	166047552	T	0.063	0.361	G	2.8E-05	NA	0.003	0.004
2	*SCN1A*	rs1021999648	166049124	T	0.063	0.361	TACTTTACAGTGCAAAGTATTTCTTCATTATGAAGAAATGACATTAGATTAGATACAGTGCAAAGATACTTTAGAC*	2.8E-05	NA	0.003	0.004
2	*SCN1A*	rs1187504368	166037655	CTT	0.065	0.361	CT	4.0E-05	NA	0.004	0.005
2	*SCN1A*	rs1541783	166055076	C	0.050	0.319	T	1.1E-04	NA	0.008	0.016
2	*SCN1A*	rs1019723	166039251	A	0.156	0.444	G	2.8E-04	NA	0.020	0.039

2	*SCN1A*	rs367905968	166052619	A	0.594	0.319	AT	1.3E-03	NA	0.085	0.178
2	*SCN1A*	rs6432861	166046718	C	0.563	0.319	T	4.3E-03	NA	0.119	0.467
2	*SCN1A*	rs13383628	166047150	T	0.563	0.319	C	4.3E-03	NA	0.119	0.467
2	*SCN1A*	rs11690959	166047515	G	0.563	0.319	A	4.3E-03	NA	0.119	0.467
2	*SCN1A*	rs1542484	166048865	A	0.563	0.319	G	4.3E-03	NA	0.119	0.467
2	*SCN1A*	rs1542483	166049062	T	0.563	0.319	A	4.3E-03	NA	0.119	0.467
2	*SCN1A*	rs1841546	166052594	C	0.563	0.319	T	4.3E-03	NA	0.119	0.467
2	*SCN1A*	rs3812718	166053034	C	0.563	0.319	T	4.3E-03	NA	0.119	0.467
2	*SCN1A*	rs2217199	166053185	A	0.563	0.319	G	4.3E-03	NA	0.119	0.467
2	*SCN1A*	rs66512822	166054359	ATGTG	0.563	0.319	A	4.3E-03	NA	0.119	0.467
2	*SCN1A*	rs1461203	166056247	C	0.563	0.319	T	4.3E-03	NA	0.119	0.467
2	*SCN1A*	rs7564306	166058385	A	0.563	0.319	G	4.3E-03	NA	0.119	0.467
2	*SCN1A*	rs6147014	166039198	C	0.469	0.236	CTGAG	4.4E-03	NA	0.119	0.483
2	*SCN1A*	rs6706163	166051520	C	0.469	0.236	A	4.4E-03	NA	0.119	0.483
2	*SCN1A*	rs6750294	166051603	A	0.469	0.236	T	4.4E-03	NA	0.119	0.483

2	*SCN1A*	rs8191987	166058504	G	0.469	0.236	A	4.4E-03	NA	0.119	0.483
22	*SHANK3*	rs148502223	50676362	T	0.078	0.000	C	1.6E-02	NA	0.403	0.930
2	*SCN1A*	rs79990586	166038417	C	0.433	0.236	T	1.6E-02	0.030	0.403	0.940
22	*SHANK3*	rs12483981	50703953	A	0.297	0.139	G	2.5E-02	NA	0.599	0.987
21	*DYRK1A*	rs17229402	37472458	A	0.063	0.000	T	3.1E-02	NA	0.610	0.998
3	*CADPS*	rs1452075	62495388	C	0.422	0.250	T	3.3E-02	NA	0.610	0.999
2	*SCN2A*	rs2304010	165367537	A	0.375	0.556	G	3.5E-02	0.014	0.610	0.999
2	*SCN2A*	rs10930160	165367696	A	0.375	0.556	G	3.5E-02	0.014	0.610	0.999
2	*SCN2A*	rs2304012	165370468	G	0.375	0.556	A	3.5E-02	0.014	0.610	0.999
2	*SCN2A*	rs767942624	165380360	T	0.375	0.556	TG	3.5E-02	0.014	0.610	0.999
2	*SCN2A*	rs12614399	165293790	C	0.297	0.472	G	3.6E-02	NA	0.610	0.999
2	*SCN2A*	rs12692768	165372999	T	0.188	0.069	C	3.8E-02	NA	0.610	1.000
2	*SCN2A*	rs1368238	165375124	G	0.188	0.069	A	3.8E-02	NA	0.610	1.000
2	*SCN2A*	rs2304015	165307838	A	0.141	0.042	G	4.2E-02	NA	0.610	1.000
2	*SCN2A*	rs62174667	165342016	G	0.219	0.375	A	4.7E-02	NA	0.610	1.000

CHR, Chromosome; dbSNP, reference SNP ID; BP, Physical position (base-pair); A1, Minor allele name (based on whole sample); F_A, Frequency of this allele in cases; F_U Frequency of this allele in controls; A2, Major allele name; P, Asymptotic *p*-value for this test; P_dom, under dominant model; FDR_P, FDR *p*-value; FAM_perm_P, Family-wise permutation test *p*-value; NA, not applicable. * Common alleles are T>C.

Supplementary Table S5. Association analysis of genotype and high-functioning autism

CHR	Gene	SNP	BP	A1	F_A	F_U	A2	P
2	*SCN1A*	rs28663047	166052559	C	0.052	0.375	T	1E-05
2	*SCN1A*	rs11691603	166035836	G	0.052	0.361	A	3E-05
2	*SCN1A*	rs1187504368	166037655	CTT	0.052	0.361	CT	3E-05
2	*SCN1A*	rs10168027	166039309	G	0.052	0.361	A	3E-05
2	*SCN1A*	rs10198801	166041507	T	0.052	0.361	G	3E-05
2	*SCN1A*	rs67636132	166045387	A	0.052	0.361	AG	3E-05
2	*SCN1A*	rs11690962	166047552	T	0.052	0.361	G	3E-05
2	*SCN1A*	rs1021999648	166049124	T	0.052	0.361	TACTTTACAGTGCAAAGTATT TCTTCATTATGAAGAAATGAC ATTAGATTAGATACAGTGCAA AGATACTTTAGAC	3E-05
2	*SCN1A*	rs1541783	166055076	C	0.037	0.319	T	8E-05

2	*SCN1A*	rs1019723	166039251	A	0.155	0.444	G	4E-04
2	*SCN1A*	rs367905968	166052619	A	0.569	0.319	AT	4E-03
2	*SCN1A*	rs6432861	166046718	C	0.552	0.319	T	8E-03
2	*SCN1A*	rs13383628	166047150	T	0.552	0.319	C	8E-03
2	*SCN1A*	rs11690959	166047515	G	0.552	0.319	A	8E-03
2	*SCN1A*	rs1542484	166048865	A	0.552	0.319	G	8E-03
2	*SCN1A*	rs1542483	166049062	T	0.552	0.319	A	8E-03
2	*SCN1A*	rs1841546	166052594	C	0.552	0.319	T	8E-03
2	*SCN1A*	rs3812718	166053034	C	0.552	0.319	T	8E-03
2	*SCN1A*	rs2217199	166053185	A	0.552	0.319	G	8E-03
2	*SCN1A*	rs66512822	166054359	ATGTG	0.552	0.319	A	8E-03
2	*SCN1A*	rs1461203	166056247	C	0.552	0.319	T	8E-03
2	*SCN1A*	rs7564306	166058385	A	0.552	0.319	G	8E-03
2	*SCN1A*	rs6147014	166039198	C	0.448	0.236	CTGAG	1E-02

2	*SCN1A*	rs6706163	166051520	C	0.448	0.236	A	1E-02
2	*SCN1A*	rs6750294	166051603	A	0.448	0.236	T	1E-02
2	*SCN1A*	rs8191987	166058504	G	0.448	0.236	A	1E-02
22	*SHANK3*	rs148502223	50676362	T	0.086	0.000	C	1E-02
2	*SCN2A*	rs2304010	165367537	A	0.345	0.556	G	2E-02
2	*SCN2A*	rs10930160	165367696	A	0.345	0.556	G	2E-02
2	*SCN2A*	rs2304012	165370468	G	0.345	0.556	A	2E-02
2	*SCN2A*	rs767942624	165380360	T	0.345	0.556	TG	2E-02
3	*CADPS*	rs1452075	62495388	C	0.448	0.250	T	2E-02
22	*SHANK3*	rs12483981	50703953	A	0.310	0.139	G	2E-02
2	*SCN2A*	rs59934051	165369848	C	0.345	0.542	G	3E-02
2	*SCN2A*	rs1252589686	165365373	G	0.357	0.556	GTATC	3E-02
2	*SCN1A*	rs79990586	166038417	C	0.411	0.236	T	3E-02
2	*SCN2A*	rs12614399	165293790	C	0.293	0.472	G	4E-02

2	*SCN2A*	rs62174667	165342016	G	0.207	0.375	A	4E-02
2	*SCN2A*	rs12692768	165372999	T	0.190	0.069	C	4E-02
2	*SCN2A*	rs1368238	165375124	G	0.190	0.069	A	4E-02
3	*SLC6A1*	rs58053962	11034823	C	0.000	0.069	G	4E-02
2	*SCN2A*	rs1469649	165370597	G	0.339	0.515	A	5E-02

CHR, Chromosome; dbSNP, reference SNP ID; BP, Physical position (base-pair); A1, Minor allele name (based on whole sample); F_A, Frequency of this allele in cases; F_U Frequency of this allele in controls; A2, Major allele name; P, Asymptotic *p*-value for this test

Supplementary Table S6. Results of association with SRS T-scores

CHR	Gene	dbSNP	BP	NMISS	BETA	SE	R2	***T***	***P***
14	*CHD8*	rs778676028	21390920	79	9.185	2.677	0.133	3.43	0.001
14	*CHD8*	rs10467770	21431472	79	9.155	2.727	0.128	3.357	0.001
2	*SCN1A*	rs10207252	166014144	79	8.738	2.766	0.115	3.159	0.002
3	*SLC6A1*	rs142733224	11028586	79	33.51	10.75	0.112	3.117	0.003
14	*CHD8*	rs12885578	21427349	77	27.52	8.895	0.113	3.094	0.003
11	*KMT5B*	rs150905417	68158751	79	30.95	10.85	0.096	2.852	0.006
11	*KMT5B*	rs138887351	68185466	79	30.95	10.85	0.096	2.852	0.006
2	*SCN1A*	rs1696715628	166038315	79	41.69	15.31	0.088	2.723	0.008
7	*CNTNAP2*	rs1462179833	146792250	79	41.69	15.31	0.088	2.723	0.008
7	*CNTNAP2*	rs919441761	148118539	79	41.69	15.31	0.088	2.723	0.008
8	*KCNQ3*	rs189917088	132185948	79	41.69	15.31	0.088	2.723	0.008
2	*SCN2A*	rs3816002	165342576	79	14.36	5.399	0.084	2.659	0.010
2	*SCN2A*	rs2304011	165370400	79	14.36	5.399	0.084	2.659	0.010
2	*SCN2A*	rs117371182	165373692	79	14.36	5.399	0.084	2.659	0.010

2	*SCN2A*	rs77089249	165377366	79	14.36	5.399	0.084	2.659	0.010
6	*ARID1B*	rs139989048	157180874	79	40.68	15.35	0.084	2.651	0.010
14	*CHD8*	rs1887546907	21391685	79	40.68	15.35	0.084	2.651	0.010
14	*CHD8*	rs189868425	21403816	79	40.68	15.35	0.084	2.651	0.010
2	*SCN1A*	rs8191989	166013988	79	-10.8	4.092	0.083	-2.638	0.010
14	*CHD8*	rs969480843	21427339	77	20.43	7.891	0.082	2.589	0.012
2	*SCN2A*	rs80044755	165367654	79	-10.05	3.985	0.076	-2.522	0.014
2	*SCN2A*	rs74798083	165386562	79	-11.77	4.674	0.076	-2.519	0.014
22	*SHANK3*	rs148502223	50676362	79	17.68	7.08	0.075	2.498	0.015
2	*SCN1A*	rs6731591	166041739	78	7.13	2.906	0.073	2.454	0.016
14	*CHD8*	rs1383083964	21427331	76	26.57	11.04	0.073	2.407	0.019
2	*SCN1A*	rs141439634	166047269	79	21.65	9.047	0.069	2.394	0.019
2	*SCN1A*	rs919198	165994670	79	-9.984	4.21	0.068	-2.371	0.020
2	*SCN1A*	rs4305294	165999649	79	-9.984	4.21	0.068	-2.371	0.020

14	*CHD8*	rs185714345	21430620	79	25.82	11.02	0.067	2.342	0.022
21	*DYRK1A*	rs16995167	37472509	79	25.82	11.02	0.067	2.342	0.022
21	*DYRK1A*	rs73903997	37473048	79	25.82	11.02	0.067	2.342	0.022
21	*DYRK1A*	rs16995170	37473153	79	25.82	11.02	0.067	2.342	0.022
22	*SHANK3*	rs139076185	50684673	79	25.31	11.04	0.064	2.292	0.025
3	*SLC6A1*	rs62238224	11034304	79	-13.17	5.748	0.064	-2.291	0.025
3	*FOXP1*	rs939845	71198106	79	10.81	4.839	0.061	2.233	0.028
6	*ARID1B*	rs287928	156829011	79	-5.461	2.512	0.058	-2.174	0.033
3	*SLC6A1*	rs2928078	11018161	79	-5.124	2.376	0.057	-2.156	0.034
12	*GRIN2B*	rs34315573	13866194	79	11.6	5.484	0.055	2.115	0.038
2	*SCN1A*	rs11691603	166035836	79	-6.615	3.192	0.053	-2.072	0.042
2	*SCN1A*	rs10168027	166039309	79	-6.615	3.192	0.053	-2.072	0.042
2	*SCN1A*	rs10198801	166041507	79	-6.615	3.192	0.053	-2.072	0.042
2	*SCN1A*	rs67636132	166045387	79	-6.615	3.192	0.053	-2.072	0.042

2	*SCN1A*	rs11690962	166047552	79	-6.615	3.192	0.053	-2.072	0.042
2	*SCN1A*	rs1021999648	166049124	79	-6.615	3.192	0.053	-2.072	0.042
2	*SCN1A*	rs1187504368	166037655	78	-6.592	3.227	0.052	-2.043	0.045
12	*GRIN2B*	rs117049493	13571511	79	12.41	6.147	0.050	2.019	0.047
2	*SCN2A*	.	165328203	79	-22.4	11.12	0.050	-2.015	0.047
6	*ARID1B*	rs142628387	157084537	79	-22.4	11.12	0.050	-2.015	0.047
7	*CNTNAP2*	rs7794448	146792274	79	5.971	2.968	0.050	2.012	0.048

CHR, Chromosome; dbSNP, reference SNP ID; BP, Physical position (base-pair); NMISS, Number of non-missing genotypes; BETA, Regression coefficient; SE, Standard error; R2, Regression r-squared; T, Wald test (based on t-distribution); P, Wald test asymptotic *p*-value

Supplementary Table S7. Results of association with IQ scores

CHR	Gene	dbSNP	BP	NMISS	BETA	SE	R2	*T*	*P*	iq_BH
21	*DYRK1A*	rs2148613619	37490571	76	29.68	6.677	0.211	4.445	3E-05	0.004
21	*DYRK1A*	.	37490572	76	29.68	6.677	0.211	4.445	3E-05	0.004
21	*DYRK1A*	.	37490573	76	29.68	6.677	0.211	4.445	3E-05	0.004
21	*DYRK1A*	.	37490574	76	29.68	6.677	0.211	4.445	3E-05	0.004
21	*DYRK1A*	.	37490585	76	29.68	6.677	0.211	4.445	3E-05	0.004
21	*DYRK1A*	rs903383174	37490565	76	28.98	7.449	0.170	3.89	2E-04	0.022
21	*DYRK1A*	.	37490567	76	28.98	7.449	0.170	3.89	2E-04	0.022
2	*SCN2A*	rs1867864	165323140	76	-9.239	2.899	0.121	-3.187	2E-03	0.169
2	*SCN2A*	rs12993173	165326704	76	-9.239	2.899	0.121	-3.187	2E-03	0.169
3	*SLC6A1*	rs113688609	11028901	76	19.15	6.646	0.101	2.881	5E-03	0.264
3	*SLC6A1*	rs112288484	11029548	76	19.15	6.646	0.101	2.881	5E-03	0.264
6	*ARID1B*	rs1269143476	157148525	76	48.31	16.88	0.100	2.862	5E-03	0.264
7	*CNTNAP2*	rs763462874	147486229	76	48.31	16.88	0.100	2.862	5E-03	0.264
22	*SHANK3*	rs757572910	50721621	76	48.31	16.88	0.100	2.862	5E-03	0.264

22	*SHANK3*	rs1291912065	50722676	76	48.31	16.88	0.100	2.862	5E-03	0.264
21	*DYRK1A*	rs17229402	37472458	76	-20.08	7.145	0.096	-2.811	6E-03	0.286
2	*SCN2A*	rs2304015	165307838	76	-13.36	4.992	0.088	-2.676	9E-03	0.357
3	*SLC6A1*	rs142509600	11034320	76	-32.18	12.1	0.087	-2.66	1E-02	0.357
3	*FOXP1*	rs187666567	70959396	76	-31.66	12.11	0.085	-2.613	1E-02	0.357
3	*FOXP1*	rs922502881	71064935	76	-31.66	12.11	0.085	-2.613	1E-02	0.357
10	*PTEN*	rs1860610300	87960830	76	-31.66	12.11	0.085	-2.613	1E-02	0.357
10	*PTEN*	rs149645874	87961340	76	-31.66	12.11	0.085	-2.613	1E-02	0.357
3	*SLC6A1*	rs35736058	11025897	76	14.86	6.374	0.068	2.331	2E-02	0.607
2	*SCN2A*	rs559599812	165370000	76	-39.85	17.17	0.068	-2.321	2E-02	0.607
6	*ARID1B*	rs9372028	156935176	76	6.437	2.777	0.068	2.318	2E-02	0.607
22	*SHANK3*	rs182778068	50704375	76	-22.9	10.06	0.065	-2.276	3E-02	0.607
2	*SCN2A*	rs2304017	165331125	76	-9.164	4.125	0.063	-2.221	3E-02	0.607
2	*SCN2A*	s3816000	165367595	76	-9.164	4.125	0.063	-2.221	3E-02	0.607

2	*SCN2A*	rs6738837	165367665	76	-9.164	4.125	0.063	-2.221	3E-02	0.607
2	*SCN2A*	rs2304014	165373420	76	-9.164	4.125	0.063	-2.221	3E-02	0.607
2	*SCN1A*	rs11691603	166035836	76	7.757	3.582	0.060	2.165	3E-02	0.607
2	*SCN1A*	rs10168027	166039309	76	7.757	3.582	0.060	2.165	3E-02	0.607
2	*SCN1A*	rs10198801	166041507	76	7.757	3.582	0.060	2.165	3E-02	0.607
2	*SCN1A*	rs67636132	166045387	76	7.757	3.582	0.060	2.165	3E-02	0.607
2	*SCN2A*	rs11690962	166047552	76	7.757	3.582	0.060	2.165	3E-02	0.607
2	*SCN1A*	rs1021999648	166049124	76	7.757	3.582	0.060	2.165	3E-02	0.607
2	*SCN2A*	rs143785800	165311859	76	-26.53	12.28	0.059	-2.16	3E-02	0.607
2	*SCN1A*	rs28663047	166052559	76	7.094	3.302	0.059	2.148	3E-02	0.607
3	*SLC6A1*	rs1238050862	11026166	76	-21.51	10.1	0.058	-2.129	4E-02	0.607
14	*CHD8*	rs1013741866	21427292	74	21.51	10.11	0.059	2.127	4E-02	0.607
2	*SCN2A*	rs7560348	165310656	76	5.414	2.6	0.055	2.083	4E-02	0.607
2	*SCN1A*	rs1541783	166055076	74	7.585	3.646	0.057	2.081	4E-02	0.607

3	*SLC6A1*	rs77090275	11029558	76	8.785	4.263	0.054	2.061	4E-02	0.607
2	*SCN2A*	rs2304016	165311993	76	-9.744	4.73	0.054	-2.06	4E-02	0.607
2	*SCN2A*	rs3769949	165342774	74	-6.104	2.963	0.056	-2.06	4E-02	0.607
2	*SCN2A*	rs12614399	165293790	76	5.22	2.557	0.053	2.041	4E-02	0.607
14	*CHD8*	rs10467770	21431472	76	-6.645	3.285	0.052	-2.022	5E-02	0.607
2	*SCN1A*	rs1187504368	166037655	75	7.144	3.547	0.053	2.014	5E-02	0.607
6	*ARID1B*	rs1331117671	156935318	76	-34.79	17.32	0.052	-2.008	5E-02	0.607
3	*SLC6A1*	rs2246543	11033817	76	8.328	4.161	0.051	2.001	5E-02	0.607

CHR, Chromosome; dbSNP, reference SNP ID; BP, Physical position (base-pair); NMISS, Number of non-missing genotypes; BETA, Regression coefficient; SE, Standard error; R2, Regression r-squared; T, Wald test (based on t-distribution); P, Wald test asymptotic *p*-value; iq_BH, FDR *p*-value

3 References

Constantino, J., and Gruber, J. (2005). Social Responsiveness Scale (SRS) Manual. Los Angeles: Western Psychological Services.

Constantino, J.N., Davis, S.A., Todd, R.D., Schindler, M.K., Gross, M.M., Brophy, S.L., et al. (2003). Validation of a brief quantitative measure of autistic traits: comparison of the social responsiveness scale with the autism diagnostic interview-revised. J. Autism Dev. Disord. 33, 427-433. doi: 10.1023/a:1025014929212.

Hus, V., Bishop, S., Gotham, K., Huerta, M., and Lord, C. (2013). Factors influencing scores on the social responsiveness scale. J. Child Psychol. Psychiatry 54, 216-224. doi: 10.1111/j.1469-7610.2012.02589.x.

Purcell, S., Neale, B., Todd-Brown, K., Thomas, L., Ferreira, M.A., Bender, D., et al. (2007). PLINK: a tool set for whole-genome association and population-based linkage analyses. Am. J. Hum. Genet. 81, 559-575. doi: 10.1086/51979.
